# Utilization of a Terrestrial Laser Scanner for the Calibration of Mobile Mapping Systems

**DOI:** 10.3390/s17030474

**Published:** 2017-02-27

**Authors:** Seunghwan Hong, Ilsuk Park, Jisang Lee, Kwangyong Lim, Yoonjo Choi, Hong-Gyoo Sohn

**Affiliations:** 1 School of Civil and Environmental Engineering, Yonsei University, Seodaemun-gu, Seoul 03722, Korea; hotaeim@yonsei.ac.kr (S.H.); moncher@yonsei.ac.kr (I.P.); ontheground@yonsei.ac.kr (J.L.); yoonjo15@yonsei.ac.kr (Y.C.); 2 Department of Computer Science, Yonsei University, Seodaemun-gu, Seoul 03722, Korea; kylim@yonsei.ac.kr

**Keywords:** mobile mapping system, terrestrial laser scanner, calibration, boresight, lever-arm

## Abstract

This paper proposes a practical calibration solution for estimating the boresight and lever-arm parameters of the sensors mounted on a Mobile Mapping System (MMS). On our MMS devised for conducting the calibration experiment, three network video cameras, one mobile laser scanner, and one Global Navigation Satellite System (GNSS)/Inertial Navigation System (INS) were mounted. The geometric relationships between three sensors were solved by the proposed calibration, considering the GNSS/INS as one unit sensor. Our solution basically uses the point cloud generated by a 3-dimensional (3D) terrestrial laser scanner rather than using conventionally obtained 3D ground control features. With the terrestrial laser scanner, accurate and precise reference data could be produced and the plane features corresponding with the sparse mobile laser scanning data could be determined with high precision. Furthermore, corresponding point features could be extracted from the dense terrestrial laser scanning data and the images captured by the video cameras. The parameters of the boresight and the lever-arm were calculated based on the least squares approach and the precision of the boresight and lever-arm could be achieved by 0.1 degrees and 10 mm, respectively.

## 1. Introduction

With the increasing demand for 3-dimensional (3D) geospatial information in various fields such as civil engineering and construction [1,2], environmental monitoring [3,4], and disaster management [5,6], a number of devices and algorithms for the 3D reconstruction have been developed and utilized. In general, all 3D mapping techniques can be classified into range-based techniques using a 3D laser scanner, also called Light Detecting and Ranging (LiDAR), and image-based 3D reconstruction techniques based on the principles of computer vision and photogrammetry [7,8]. By using those techniques, the 3D information (X, Y, Z) of observed objects is represented by a point cloud. The 3D laser scanner directly measures the 3D coordinates of objects with extremely high accuracy and resolution, but is financially prohibitive. Alternatively, image-based 3D reconstruction methods have been developed and applied to reducing the cost of acquiring point clouds [9,10]. Using corresponding features in overlapped images, the 3D coordinates of objects are calculated. However, the image-based 3D reconstruction technique has much noise and low accuracy and is highly affected by the captured space. If there is no feature in the captured space or the space is too dark or bright, the 3D reconstruction cannot be performed and many noisy points occur. In this regard, the 3D laser scanner is utilized by the engineers who require geospatial data of high resolution and accuracy [11,12].

According to the scanning geometry, laser scanners can be classified into terrestrial laser scanners and mobile laser scanners. Terrestrial laser scanners spherically scan the surrounding space using two freely rotating axes in a fixed position and generate more accurate, precise, and dense information than mobile laser scanners [13]. With their high performance, terrestrial laser scanners has been widely applied to the fields requiring highly accurate and dense 3D information like construction sites [14,15]. On the other hand, mobile laser scanners mounted on a moving vehicle rapidly rotate or oscillate horizontally at a certain fixed vertical angle. In general, mobile laser scanners with high scan rates are designed to be mounted on different vehicles such as automobiles and drones. As the platform where the mobile laser scanner is mounted moves, a point cloud is generated with respect to the trajectory of the platform. The trajectory information can be obtained from a navigation sensor like a Global Navigation Satellite System (GNSS)/Inertial Navigation System (INS) and the point cloud is formed based on the geometric relationship between the navigation sensor and the laser scanner [13,16]. Moreover, by integrating the mobile laser scanner with a camera, color information can be added to the generated point cloud.

A system combining multiple sensors with a navigation sensor on a moving vehicle is called a Mobile Mapping System (MMS). In the early 1990s, MMSs combining a code-only GNSS, stereo digital cameras, and supplementary dead-reckoning sensors were developed and utilized in applications based on the image-based 3D reconstruction technique [17,18]. As the accuracy of laser measurement and navigation sensors has improved, mobile laser scanners have become one of the main components of any MMS. In particular, as near real-time and periodical 3D mapping is required for the autonomous driving systems, the laser-based measurement MMS has been developed and widely utilized to generate the high-quality 3D geospatial information about urban environments [19,20,21,22].

To integrate the datasets captured by each sensor mounted on the MMS into the unified single coordinate system, the calibration, which is the process to estimate the orientation (boresight) and position (lever-arm) parameters, is required with the reference datasets [16,23,24]. When the boresight and lever-arm parameters defining the geometric relationship between each sensing data and GNSS/INS data are determined, georeferenced data can be generated. However, even after precise calibration, the boresight and lever-arm parameters of an MMS can be shaken and the errors that deteriorate the accuracy of the georeferenced data might accumulate. Accordingly, for the stable operation of multiple sensors, precise calibration must be conducted periodically.

In general, the calibration process is performed based on the observation models and constraints to define the geometric relationship between the observed object in real world and in the sensing data. For example, in the case of camera calibration, a calibration model is generally designed based on a collinearity equation with the Exterior Orientation Parameters (EOPs) and Interior Orientation Parameters (IOPs) [25,26]. To configure the constraints for the camera calibration, a checkerboard which has repetitive black and white (BW) patterns and whose spacing is accurately known is generally utilized [27,28]. For example, computer vision libraries such as OpenCV [29] and Matlab Toolbox [30] provide camera calibration tools based on the checkerboard approach. With the calibrated parameters, the correction of lens distortion, which is presented in a fisheye lens or wide angle lens camera image, and geometric analysis such as visual odometry can be performed [31]. Furthermore, ground control features of which ground coordinates are known can be used as geometric constraints. For example, since the checkerboard-based calibration method is difficult to apply to an airborne system due to the long distance between the sensor and ground, Chiang et al. [32] used ground control points to calibrate the time-offset of the camera shutter and to estimate the trajectory of the camera mounted on an airborne vehicle.

While point-based calibration techniques using a checkerboard or ground control points can be a practical solution for the calibration of camera systems, it is difficult to extract accurate corner or edge points from the sparse point clouds generated by the mobile laser scanner due to its low accuracy and resolution. Alternatively, the line, plane, and cylindrical features, which can be defined by mathematical equations, have been widely applied for the calibration of laser scanners [33,34,35]. For example, the plane features can be precisely extracted from a sparse point cloud using a RANdom SAmple Consensus (RANSAC) algorithm even if there exist noisy points in the point cloud [36]. With the orthogonality constraints of multiple planes installed in the laser scanning view, the boresight parameters can be estimated with least-squares adjustment [37,38,39,40]. Furthermore, based on the least-squares adjustment, the boresight and lever-arm parameters of the MMS can be stochastically calculated using the plane features as geometric constraints. Filin [41] applied the least-squares adjustment with the plane features to calibrate an airborne laser scanning system. Glennie [42] performed the boresight calibration of a mobile laser scanner with the plane features captured in a kinematic mode.

Obviously, the precision of the calibrated parameters is directly affected by the accuracy and geometry of the ground control features, and the construction of the accurate and dense ground control features for the calibration is important. To collect the ground control features, a total station or a laser tracker, which is the laser-based equipment to achieve the 3D coordinate of a point target with a sub-millimeter accuracy, are generally utilized [43,44,45]. Even though the observation accuracy of the total station is significantly high, the point positioning techniques are labor-intensive and it is difficult for general users to achieve a number of accurate control point coordinates. Moreover, since different types of features are required for the calibration of each sensor on the MMS, it is difficult to make a common ground control dataset.

In this paper, we have devised a method for utilizing the terrestrial laser scanner to simultaneously calibrate the camera and mobile laser scanner mounted on the MMS. On our MMS, devised for conducting the calibration experiment, three network video cameras, one mobile laser scanner, and one GNSS/INS were mounted. The devised MMS calibration process can be largely divided into two steps. As the first step for constructing the dataset of the ground control features, the terrestrial laser scanning data needs to be accurately georeferenced. In the second step point and plane features were extracted from the georeferenced terrestrial laser scanning data and matched with the features extracted from the mobile laser scanning data and the captured images. Before applying the boresight and lever-arm calibration of the devised MMS, the camera calibration to estimate the camera IOPs was conducted separately using the checkerboard approach. The calibration parameters of each sensor and their precisions were calculated based on the least-squares adjustment.

## 2. Methodology

### 2.1. Overview

On the MMS, three network video cameras, a mobile laser scanner, and a GNSS/INS were mounted and combined with a steel-welded frame to fix each sensor. The MMS was designed for two purposes; (a) generation of the point cloud including color information of scanned areas; and (2) 3D mapping of the objects extracted from images.

**Figure 1:**
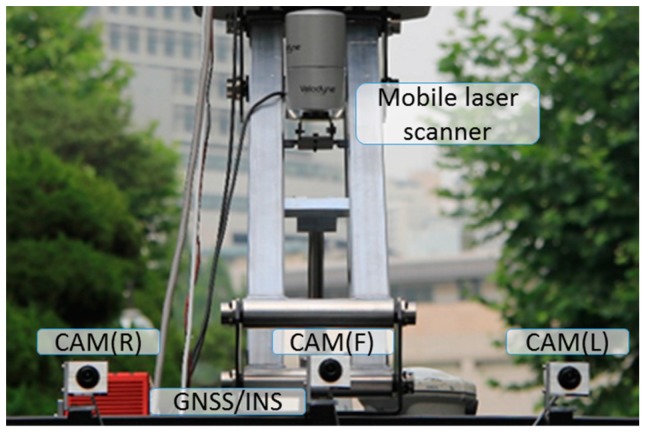
Configuration of mobile mapping system: network video cameras (F: front, L: left, R: right), mobile laser scanner, and GNSS/INS.

Figure 1 illustrates the MMS which was developed and applied for the experiments verifying the proposed calibration approach. To utilize the terrestrial laser scanning data as a reference source data for calibration, the post-processing of the terrestrial laser scanning data was conducted in two steps namely: (1) registration, which is to merge multiple point clouds into a common point cloud, and (2) georeferencing, which is to convert the relative coordinate system of point clouds into an absolute coordinate system.

Before the boresight and lever-arm calibration of the MMS, the camera calibration of each camera sensor was conducted to define the accurate geometry of the cameras by estimating IOPs. The camera calibration algorithm was designed by the collinearity equation including the IOPs, and a checkerboard was utilized to conduct the camera calibration.

After processing the terrestrial laser scanning data and estimating the camera IOPs, the boresight and lever-arm calibration was conducted. To conduct this calibration, reference features were extracted from the datasets of the video cameras, the mobile laser scanner, and the terrestrial laser scanner. Point features were extracted for the calibration of the camera sensors and the plane features were extracted for the calibration of the mobile laser scanner.

**Figure 2:**
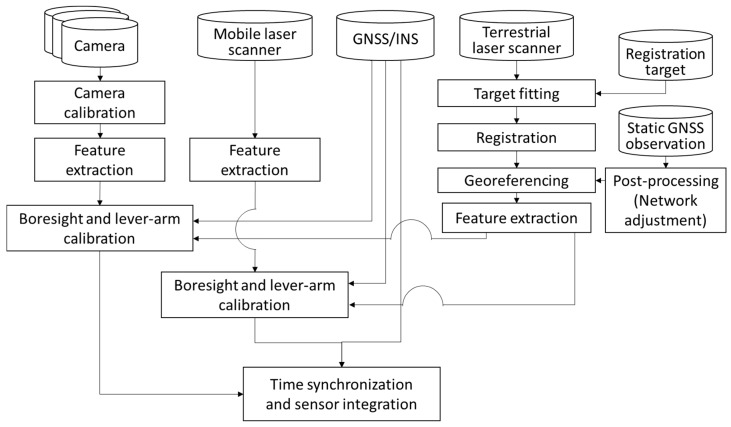
Sensor calibration and integration scheme of mobile mapping system.

Based on the parameters estimated from the calibration process, the integration of multiple sensors mounted on the MMS were conducted. Moreover, since each sensor is conducting data sampling in its own time frame, the time synchronization among the sensors was performed based on the time-dependent linear interpolation with respect to the position and orientation of the MMS platform. The overall process of our sensor calibration and integration method of the MMS is depicted in Figure 2.

### 2.2. Camera Calibration

The camera sensor captures an image by collecting the rays reflected from targets. When the camera sensor receives rays through its lens, the geometry between coordinates of observed targets and image can be represented by the collinearity equation including the parameters of image coordinates (*x_i_*,*y_i_*), focal length (*c*), principal point (*x_p_*,*y_p_*), lens distortion (Δ*x_i_*,Δ*y_i_*), camera position (*x_c_*,*y_c_,z_c_*), camera orientation (*m*_11_ ~ *m*_33_), and object position (*x_o_*,*y_o_,z_o_*). The collinearity equation can be represented by Equation (1) [25]:

(1) $$\begin{array}{l} x_{i}=x_{p}-x_{n}+\Delta x_{i}\\ y_{i}=y_{p}-y_{n}+\Delta y_{i} \end{array}$$

where, *x_n_*,*y_n_* can be calculated by Equation (2), and the camera orientation parameters (*m*_11_ ~ *m*_33_) can be calculated from the rotation angle (*ω*,*ϕ*,*κ*) by Equation (3):

(2) $$x_{n}=-c\frac{m_{11}(x_{o}-x_{c})+m_{12}(y_{o}-y_{c})+m_{13}(z_{o}-z_{c})}{m_{31}(x_{o}-x_{c})+m_{32}(y_{o}-y_{c})+m_{33}(z_{o}-z_{c})}\;y_{n}=-c\frac{m_{21}(x_{o}-x_{c})+m_{22}(y_{o}-y_{c})+m_{23}(z_{o}-z_{c})}{m_{31}(x_{o}-x_{c})+m_{32}(y_{o}-y_{c})+m_{33}(z_{o}-z_{c})}$$

(3) $$\left[\begin{array}{ccc} m_{11} & m_{12} & m_{13}\\ m_{21} & m_{22} & m_{23}\\ m_{31} & m_{32} & m_{33} \end{array}\right]=\left[\begin{array}{ccc} \cos\varphi \cos\kappa & \cos\omega \sin\kappa +\sin\omega \sin\varphi \cos\kappa & \sin\omega \sin\kappa -\cos\omega \sin\varphi \cos\kappa \\ -\cos\varphi \sin\kappa & \cos\omega \cos\kappa -\sin\omega \sin\varphi \sin\kappa & \sin\omega \cos\kappa +\cos\omega \sin\varphi \sin\kappa \\ \sin\varphi & -\sin\omega \cos\varphi & \cos\omega \cos\varphi \end{array}\right]$$

Moreover, the lens distortion is generally modelled by radial distortion and tangential distortion and can be represented by Equation (4):

(4) $$\begin{array}{l} \Delta x_{i}=x_{n}(A_{1}r_{n}^{2}+A_{2}r_{n}^{4}+A_{3}r_{n}^{6})+B_{1}(r_{n}^{2}+2x_{n}^{2})+2B_{2}x_{n}y_{n}\\ \Delta y_{i}=y_{n}(A_{1}r_{n}^{2}+A_{2}r_{n}^{4}+A_{3}r_{n}^{6})+2B_{1}x_{n}y_{n}+B_{2}(r_{n}^{2}+2y_{n}^{2}) \end{array}$$

where, *r_n_* is $\sqrt{x_{n}^{2}+y_{n}^{2}}$, *A*_1_, *A*_2_ and *A*_3_ are the radial distortion parameters and *B*_1_, *B*_2_ are the tangential distortion parameters.

In general, the IOPs in the collinearity equation can be obtained from a camera specification but geometric errors exist in the image measurement system. To define the mathematical relationship among the sensors mounted on the MMS, accurate IOPs must be calculated by means of the camera calibration [44]. In this paper, a checkerboard with 30 mm spacing was used for the camera calibration. Fifteen images in different positions and angles were captured from each camera sensor to achieve the sufficient geometry to establish the correlations among parameters [46].

### 2.3. Registration and Georeferencing of Terrestrial Laser Scanning Data

To utilize terrestrial laser scanning data as reference data for the MMS calibration, the registration and the georeferencing of the scanning data were sequentially conducted. Basically, both processes are for estimating the rotation and the translation parameters with respect to the reference data using corresponding features or points, and the algorithms for the boresight and lever-arm calibration are similar. In registration case, one of the point clouds among the observation data at multiple stations is set as the reference. On the other hand, for the georeferencing, the points in an absolute coordinate system are utilized. In this paper, the Geodetic Reference System (GRS) 80 Korean Transverse Mercator (TM) coordinate system, which is an official legal system of South Korea [47], was applied as the absolute coordinate system. The origin is 127°00’ east longitude, 38°00’ north latitude, the scale factor is 1, the false northing is 600,000 m, and the false easting is 200,000 m.

**Figure 3:**
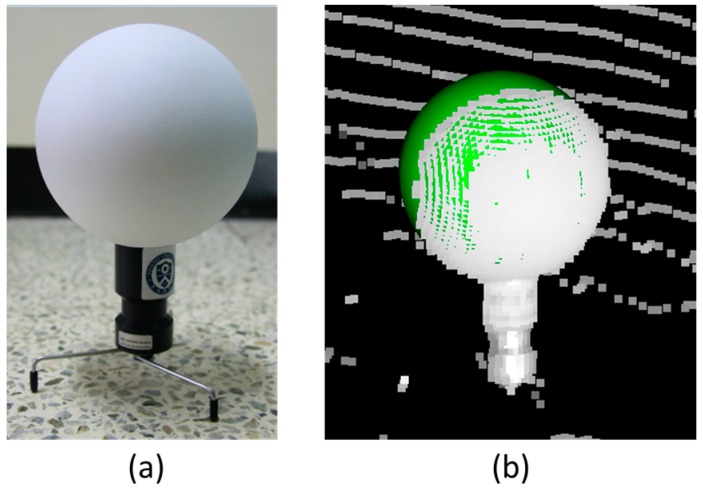
(**a**) Sphere target used for registration of terrestrial laser scanning data; (**b**) sphere target detected in a point cloud (the green sphere is a fitted sphere model).

The registration can be categorized into the target-based registration using artificial targets and the target-free registration based on minimizing locational discrepancy among point clouds. In general, the target-based registration using paper, paddle, and sphere targets is applied for the application requiring high accuracy and the target-free registration has uncertainty according to the shape and quality of the observed point clouds [48]. In particular, Becerik-Gerber et al. [49] have demonstrated that a sphere target has the highest precision in registration. For this reason, sphere targets whose diameters were 145 mm were utilized. Figure 3 illustrates these sphere targets.

As shown in Figure 3b, the sphere targets can be detected in a point cloud and fitted to a mathematical model. Through the fitted model, the center point of the sphere can be calculated and used as the control point to transform multiple point clouds. The registration targets must be fixed during scanning and a sufficient number of the targets must be installed in the overlapped scan areas. Paper and paddle targets also can be used for the registration. However, since the quality of the observed point cloud is affected by the incidence angle, the geometry among the targets and scanner should be designed carefully [49,50]. Moreover, the target-free registration, also called the Iterative Closest Point (ICP), can be additionally applied to improve the precision of the registration but the approach might have uncertainty in the registration results [4,51]. The occlusions and insufficient geometric constraints in the point clouds might cause errors in the registration process.

**Figure 4:**
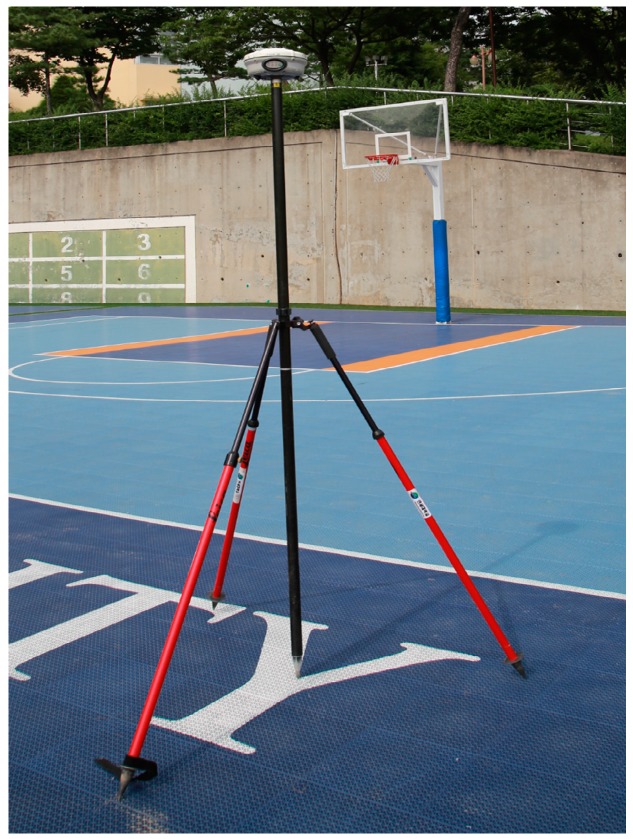
Static GNSS observation conducted for georeferencing of terrestrial laser scanning data.

After the registration process, the georeferencing process is conducted to convert the relative coordinate system of the point cloud into an absolute coordinate system. For the georeferencing, control points with known absolute 3D positions are required. The static GNSS technique using Trimble’s R8 instrument, which can obtain positioning accuracy of millimeter-level, was applied to obtain the control points in this paper. The network adjustment based on the base stations managed by the Korean National Geographic Information Institute was conducted for the post-processing of the GNSS observation [52]. Figure 4 shows the static GNSS observation conducted for the georeferencing of the terrestrial laser scanning data.

### 2.4. Mobile Mapping System Calibration

**Figure 5:**
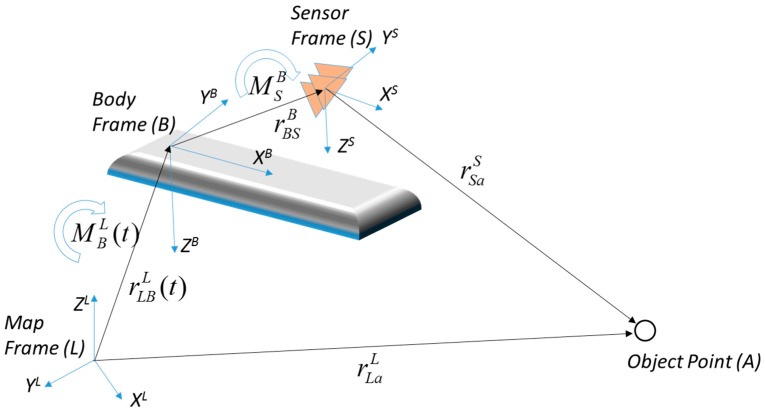
Conceptual model of mobile mapping system.

For the sensor integration of the MMS, the mathematical models with accurate parameters to transform each observation data into another sensor system or an absolute coordinates system must be defined, and Figure 5 describes the conceptual model of the MMS [33,43,53,54,55].

As shown in Figure 5, the geometry among the object point (*A*), sensor frame (*S*), body frame (*B*), and map frame (*L*) can be defined mathematically by the rotation and translation relationship. The body frame has the relative coordinate system to combine multiple sensors and the coordinate system of the body frame is transformed into the map frame with the position and orientation information observed by a GNSS/INS. The mathematical model for the geometric relationship can be defined by Equation (5):

(5) $$r_{La}^{L}=r_{LB}^{L}(t)+M_{B}^{L}(t)(M_{S}^{B}r_{Sa}^{S}+r_{BS}^{B})$$

where, $r_{La}^{L}$ is the coordinate of *A* in the map frame, *t* is the observation time, $r_{LB}^{L}\left(t\right)$ is the position of the body frame in the map frame, $M_{B}^{L}\left(t\right)$ is the rotation matrix from the body frame to the map frame, $M_{S}^{L}$ is the rotation matrix from the sensor frame to the body frame, $r_{Sa}^{S}$ is the position of *A* in the sensor frame, and $r_{BS}^{B}$ is the position of the sensor in the body frame.

In addition, when an object point (*A*) is projected onto the image in the camera frame (*C*), the geometric relationship includes the scale parameter (*λ_a_*) for *A*. Accordingly, the mathematical model for the camera sensor can be defined by Equation (6):

(6) $$r_{La}^{L}=r_{LB}^{L}(t)+M_{B}^{L}(t)(\lambda_{a}M_{C}^{B}r_{Ca}^{C}+r_{BC}^{B})$$

where, $M_{C}^{B}$ is the rotation matrix from the camera frame to the body frame, $r_{Ca}^{C}$ is the position of *A* in the camera frame, and $r_{BC}^{B}$ is the position of the camera in the body frame. Each sensor in the body frame has individual parameters ($M_{S}^{B}$, $r_{BS}^{B},\;M_{C}^{B},r_{BC}^{B})$ for transforming the data in the sensor frame into the body frame. Moreover, the MMS has common parameters ($r_{LB}^{L}\left(t\right),M_{B}^{L}\left(t\right))$ for transforming the data in the body frame into the map frame. The position and scale parameters of the object point ($\lambda_{a},r_{Sa}^{S},r_{Ca}^{C})$ are determined for every observed point.

### 2.5. Adjustment Model

To estimate the optimized parameters for each sensor frame, the least squares adjustment is widely applied [16,19,25,53,55,56,57]. The least squares approach is basically designed based on the mathematically defined models which is called an observation equation. For the computation, the observation equation is represented in a matrix form as Equation (7):

(7) y=Aξ+e

where, *y* is the observation vector, *A* is the design matrix, *ξ* is the unknown parameter vector (*x*_1_,*x*_2_,…,*x_n_*), *e* is the random error vector. It is assumed that the random errors follow the normal distribution ($e\sim \left(0,\sigma_{0}^{2}P^{-1}\right))$. $\sigma_{0}^{2}$ is the variance component used as scale, and *P* is the weight matrix which is the inverse matrix of the variance-covariance matrix. The weight is inversely proportional to the observation variance and the covariance between uncorrelated observations is zero. The high variance indicates that the observation has a large error and requires a large correction. When a measurement system has different precision observations, the weight matrix is controlled by the observation variance. In this paper, the weight matrix for image points was basically set as the identity matrix with the assumption that the observations have identical precisions. Furthermore, the weight matrix is controlled when features such as lines or planes in images or point clouds are utilized as the observation of the system [24,58]. For the plane features extracted from the point cloud, the precisions of the points in the normal direction of the plane were set as one, and those in the other direction were set as zero. By this approach, the similarities of the plane features in pairwise datasets can be measured and the optimized parameters which maximize the similarity and minimize the discrepancy can be estimated. Furthermore, when it is predicted that the precisions of control features are different, the weight matrix should reflect their precisions. In this paper, for the plane features extracted from laser scanning data, the weights reflected the inverses of squared plane model fitting errors.

From Equation (7), the least squares solution is designed to minimize the random error vector and find the most probable value of unknown parameters. The most probable value can be represented by Equation (8):

(8) $$\hat{\xi }={\left(A^{T}PA\right)}^{-1}A^{T}Py$$

While Equations (7) and (8) deal with the observation model which consists of linear equations, the calibration models of image and laser sensors are nonlinear. For this reason, the nonlinear systems have to be linearized with the first-order Taylor series approximation of the observation equations and the equations can be modified by Equations (9)–(11):

(9) $$y-F(\xi_{o})=J\Delta \xi +e$$

(10) $$\hat{\Delta \xi }={\left(J^{T}PJ\right)}^{-1}J^{T}P(y-F(\xi_{o}))$$

(11) $$\xi_{new}=\xi_{o}+\hat{\Delta \xi }$$

where, *ξ_o_* is the approximate parameter vector before the correction, Δ*ξ* is the correction vector of the unknown parameters, *ξ_new_* is the updated parameter vector after the correction, *F* is the nonlinear observation system with respect to *ξ_o_*, and *J* is the Jacobian matrix which includes the linearized equations of the nonlinear observation model and configured as Equation (12):

(12) $$J=\left[\begin{array}{cccc} \frac{\partial F_{1}(\xi_{o})}{\partial x_{1}} & \frac{\partial F_{1}(\xi_{o})}{\partial x_{2}} & \cdots & \frac{\partial F_{1}(\xi_{o})}{\partial x_{n}}\\ \frac{\partial F_{2}(\xi_{o})}{\partial x_{1}} & \frac{\partial F_{2}(\xi_{o})}{\partial x_{2}} & \cdots & \frac{\partial F_{2}(\xi_{o})}{\partial x_{n}}\\ \vdots & \vdots & \ddots & \vdots \\ \frac{\partial F_{m}(\xi_{o})}{\partial x_{1}} & \frac{\partial F_{m}(\xi_{o})}{\partial x_{2}} & \cdots & \frac{\partial F_{m}(\xi_{o})}{\partial x_{n}} \end{array}\right]$$

For the boresight and lever-arm calibration of an MMS, the mathematical models for the geometric relationship to convert the point coordinates in each sensor frame into the map frame were used as *F*, and *J* was derived. *ξ* consisted of the boresight and lever-arm parameters. Meanwhile, for the camera calibration, the collinearity equation was used as *F*, and *ξ* consisted of the camera IOPs. The adjustment process is iteratively conducted until Δ*ξ* is almost zero or convergent. With the assumption that the observation errors follow the normal distribution, the uncertainty of adjusted parameters is also derived based on the law of error propagation. The dispersion of the adjusted parameters can be calculated by Equation (13):

(13) $$D\left\{\hat{\Delta \xi }\right\}={\hat{\sigma }}_{o}^{2}{\left(J^{T}PJ\right)}^{-1}$$

where ${\hat{\sigma }}_{o}^{2}$ is calculated by Equation (14):

(14) $${\hat{\sigma }}_{o}^{2}=\frac{{\tilde{e}}^{T}P\tilde{e}}{n-m}$$

where, *n* is the number of observations, *m* is the number of unknown parameters, and $\tilde{e}$ is the residual vector.

With Equations (9)–(14), the least squares adjustment process can be conducted formulaically and the camera IOPs, the boresight and lever-arm parameters of the image and laser sensors in the MMS could be estimated. In the practical application of the least squares adjustment approach, the theoretically minimum number of the utilized features is basically determined by the number of the unknown parameters. To estimating the boresight and lever-arm parameters of each sensor, at least three points or four planes are required, respectively. Moreover, the geometry of the features are very important. The points must not exist in a single line and the planes must not be parallel or coincide.

### 2.6. Feature Extraction

As reference data for the boresight and lever-arm calibration, point and plane features were extracted from the images and point cloud. For the point feature extraction, the Harris corner detection algorithm was applied [59]. At the corner point in an image, the changes of the pixel values are remarkable in all direction. At a certain point (*x_i_*,*y_i_*), the variation (*E*(*x_i_*,*y_i_*)) of pixel values (*I*(*x_i_*,*y_i_*)) for the shift (Δ*x*,Δ*y*) in the window of which size is *w* can be represented by Equation (15):

(15) $$E(x_{i},y_{i})=\sum_{\Delta x=-w}^{w}\sum_{\Delta y=-w}^{w}{\left[I(x_{i}+\Delta x,y_{i}+\Delta y)-I(x_{i},y_{i})\right]}^{2}$$

where, by the Taylor expansion, Equation (15) can be approximated and arranged in a matrix form with a symmetric matrix (*M*) as Equation (16):

(16) $$E(x_{i},y_{i})\approx [\begin{array}{cc} \Delta x & \Delta y \end{array}](\sum_{\Delta x=-w}^{w}\sum_{\Delta y=-w}^{w}\left[\begin{array}{cc} I_{x}^{2} & I_{x}I_{y}\\ I_{x}I_{y} & I_{y}^{2} \end{array}\right])\left[\begin{array}{c} \Delta x\\ \Delta y \end{array}\right]=[\begin{array}{cc} \Delta x & \Delta y \end{array}]M\left[\begin{array}{c} \Delta x\\ \Delta y \end{array}\right]$$

where, *I_x_* and *I_y_* are the gradients of the pixel values in the x direction and y direction, respectively. Then, the score (*R*) for determining corner points can be calculated by Equation (17):

(17) $$R=\mathrm{determiant}(M)-k{\left(\mathrm{trace}(M)\right)}^{2}$$

where, *k* is the constant for controlling the ratio of the influence between determinant(*M*) and trace(*M*). The pixels of which windows have *R* higher than a certain threshold are classified as the corner points in the image.

**Figure 6:**
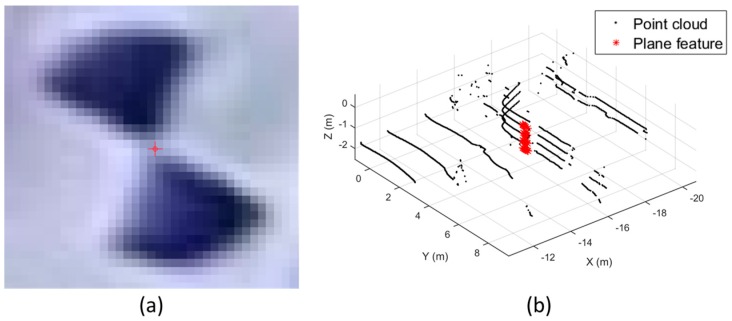
Example of reference feature extraction: (**a**) point feature extracted from image; (**b**) plane feature extracted from mobile laser scanning data.

For extracting plane features from a mobile laser scanning data, the RANSAC scheme was applied. The RANSAC scheme defines model parameters by iterative processes of hypothesis and verification. In the hypothesis step, sample points are randomly extracted from a dataset and form a plane model. Then, in the verification step, the points within the distance criterion are classified as inliers and the Root Mean Squared Error (RMSE) between the plane model and inliers is calculated as a score to adopt the best plane model. For the best results, the number of the iteration (*k*) is determined by Equation (18):

(18) $$k=\frac{\log(1-p)}{\log(1-w^{n})}$$

where, *p* is the probability that the best model is returned by *k* iterations, *w* is the probability that a point belongs to the best model, and *n* is the number of sample points. The plane model applied is defined by Equation (19):

(19) ax+by+cz+1=0

where, *a*,*b*,*c* are the parameters of the plane model, and *x,y,z* are the 3D coordinates of a point. Figure 6 describes the examples of the point and plane features extracted from each sensing data. As shown in Figure 6b, the point cloud obtained by the mobile laser scanner is too sparse to extract the point features. Therefore, plane features were extracted and applied on the boresight and lever-arm calibration of the mobile laser scanner.

## 3. Data Preparation

### 3.1. Sensors

To obtain continuous multi-view stereo images, three AXIS F1005-E, which are network video cameras were combined [60]. The effective sensor size of the camera is 1/2.8″, and the size of the captured image is 1920 × 1200 pixels. The maximum frame rate is 60 fps which is sufficient for compensating the image motion blur that occurs when the sensor platform moves fast. The camera of which focal length is 10.5 mm could achieve 113° horizontal FOV and 62° vertical FOV. Table 1 summarizes the specifications of the AXIS F1005-E (AXIS Communications AB, Emdalavägen Lund, Sweden).

For 3D laser scanning of target objects, the Velodyne HDL 32-E (Velodyne, Morgan Hill, CA, USA) was adopted [61]. The mobile laser scanner has rotating 32 channels using a Class 1 laser, which is safe for general users under all conditions [62]. The maximum measurement range is up to 100 m and the rotation rate of the laser scanners varies from 5 to 20 Hz. The laser positioning accuracy is ± 2 cm. Its horizontal FOV and angular resolution are 360° and 0.1~0.4°, respectively. However, its vertical FOV are only −30.67 to 10.67° and the vertical angular resolution is 1.33°. Table 2 summarizes the specifications of the HDL 32-E.

Since the observations based on image and laser scanning are conducted on a fast-moving vehicle, the GNSS/INS which can obtain significantly precise and dense navigation information is essential, and OxTS survey+ was adopted [63]. The positioning and orientation accuracies of the GNSS/INS are 0.01 m and 0.1 degrees, respectively, and the output rate is 100 Hz. Table 3 summarizes the specifications of the OxTS survey+.

To obtain the reference data for the boresight and lever-arm calibration of the developed MMS, FARO’s Focus 3D was adopted [64]. Its maximum measurement range and scan rate are 120 m and 976,000 points/sec, respectively. The ranging error and noise are only ±2 mm and 0.6 mm, respectively. Moreover, the horizontal and vertical FOVs are 360° and 305°, respectively. Table 4 summarizes the specifications of the Focus 3D.

With the terrestrial laser scanner, dense and precise 3D information of target objects can be constructed rapidly and effectively. However, since the terrestrial laser scanner must be fixed while scanning, it is not suitable for an MMS. Furthermore, the terrestrial laser scanner can only observe objects in line of sight so occlusion might occur in the observed point cloud. To maximize the coverage of the laser scanning and minimize the occlusion, multiple scanning and registration processes have to be conducted. For the registration of each scanned data set, common targets must be set at the areas overlapped in the multiple point clouds. In this paper, the artificial sphere targets were utilized for registration. The georeferencing process was followed to transform the coordinate system of the registered point cloud into an absolute coordinate system. For the georeferencing process, the static GNSS observation which can ensure 3.5 mm accuracy was conducted using the Trimble R8 GNSS receiver [65].

### 3.2. Datasets

**Figure 7:**
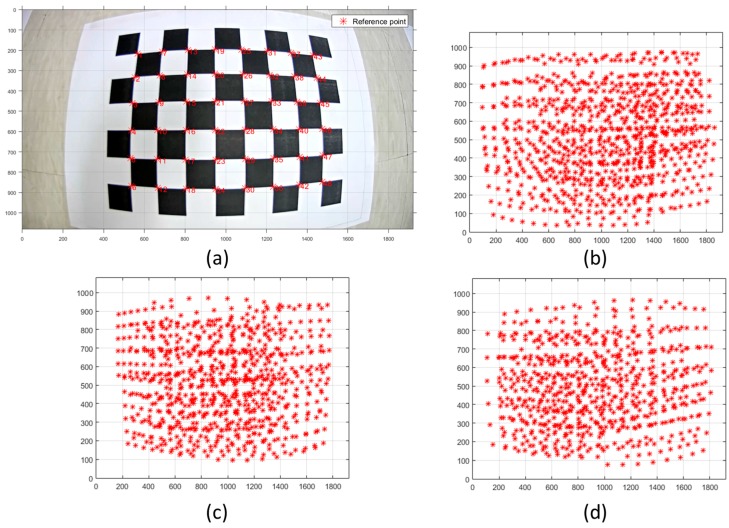
(**a**) Checkerboard and extracted reference points; (**b**) reference points for calibration of CAM(F); (**c**) reference points for calibration of CAM(L); (**d**) reference points for calibration of CAM(R).

To do the camera calibration, a checkerboard whose grid size of squares is 30 × 30 mm was utilized [66,67]. Figure 7 shows the checkerboard and extracted reference points.

As shown in Figure 7a, the reference points could be detected from the checkerboard in captured images. Moreover, as shown in Figure 7b–d, a sufficient number of the points were utilized to achieve the geometry to release the correlation between camera calibration parameters. The checker points configure the virtual grid having 30 mm spacing and the actual camera IOPs and virtual camera EOPs can be estimated by the least squares adjustment with their precision.

**Figure 8:**
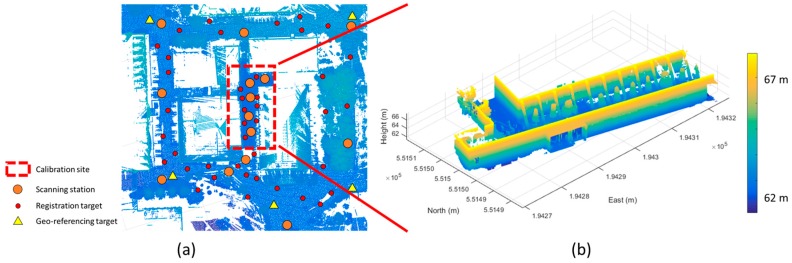
Terrestrial laser scanning data: (**a**) distribution of scanning stations, registration targets, and georeferencing targets; (**b**) registered and georeferenced point cloud of calibration site.

After the camera calibration process of each camera sensor was done, the boresight and lever-arm calibration was conducted using the terrestrial laser scanning data. Due to the limitation of the single laser scanning coverage, 15 repetitions of the terrestrial laser scanning were conducted. Moreover, the registration and georeferencing processes were performed to use the scanning data as the reference data for the boresight and lever-arm calibration of the MMS. Figure 8 describes the distribution of the scanning stations, the targets for the registration and georeferencing, and the processed point cloud which was registered into the Korean TM coordinate system.

As shown in Figure 8, the registration targets were well distributed by considering the locations of the scanning stations and the sufficient number of the georeferencing targets were installed to cover the scanned area. The point density of the point cloud in the calibration site was about 10 mm per 1 m distance from the terrestrial laser scanning station.

**Figure 9:**
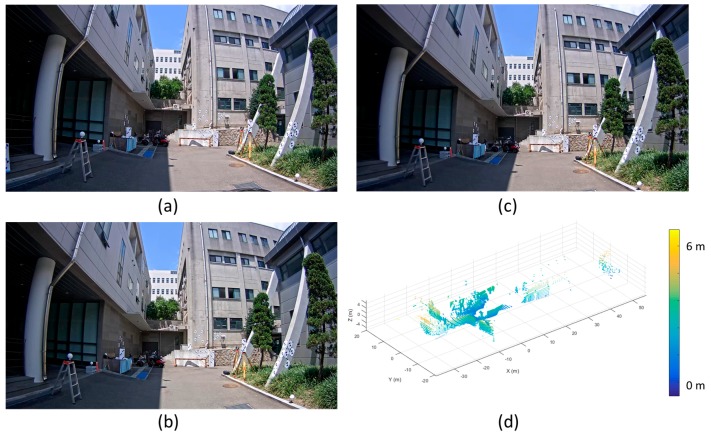
Datasets observed from image and laser sensors in MMS: (**a**) CAM(F) image; (**b**) CAM(L) image; (**c**) CAM(R) image (**d**) mobile laser scanning data (The origin of the coordinate system is center of sensor).

At the calibration site, the BW targets whose diameters were 15 cm were installed to extract the reference point and plane features from the images and the point cloud. Figure 9 describes the datasets observed by the developed MMS.

As shown in Figure 9a–c, there existed a significant amount of image distortion in each image. Furthermore, compared with the terrestrial laser scanning data (Figure 8b), the point cloud observed by the mobile HDL 32-E was notably sparse. This is because the vertical FOV of the mobile laser scanner was relatively narrow and the vertical angular resolution value was relatively wide.

From the image and point cloud data obtained from each sensor, the reference features were collected for the MMS calibration. The absolute coordinates of the center points of the BW targets were extracted from each images and the reference point cloud. To extract the image coordinates of the targets, the Harris corner detection algorithm was applied [59]. Then, the absolute 3D position of the targets were extracted from the terrestrial laser scanning data using the Scene v5.3, which is the software provided with the FARO Focus 3D [68]. To extract the plane features from each laser scanning data, the RANSAC scheme was applied.

**Figure 10:**
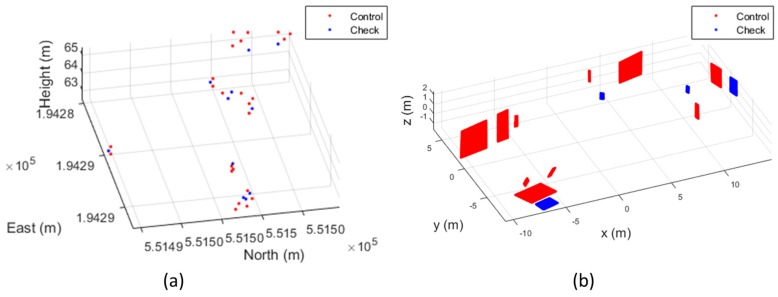
Distribution of reference features for boresight and lever-arm calibration: (**a**) point features; (**b**) plane features.

For the experiments, 35 point features and 14 plane features were extracted. The plane fitting errors were less than 5 mm for the terrestrial laser scanning data and less than 1 cm for the mobile laser scanning data. Among 49 extracted features, 25 points and 10 planes were used as the ground control features to estimate the boresight and lever-arm parameters and the others were used as the independent check features for the external check of the calibration. For the network video camera sensors, the locational errors between the coordinates of features projected onto an image and the coordinates extracted from the image were calculated. For the mobile laser scanner, the discrepancies between the mathematically defined planes and the points transformed based on the estimated parameters were calculated. Figure 10 shows the distribution of the extracted features.

## 4. Calibration Results

### 4.1. Camera Calibration Results

The camera calibration of each camera sensor was conducted using the images capturing the reference checkerboard. Moreover, the initial focal length for the iterative least squares solution approach was set up according to the specification provided by the manufacturer and refined by minimizing the projection residuals of the reference points with the estimated principal points and lens distortion parameters. Table 5 summarizes the initial values and camera calibration results. The table shows the differences between the initial values and calibrated parameters. The difference might have an influence on the boresight and lever-arm calibration results. For example, since the error in estimating the focal length means the error in the image projection depth, the error in the focal length might cause error in the lever-arm calibration results in the direction of the boresight. Furthermore, the principal points might cause the error in the lever-arm calibration results in the normal direction of the boresight. The lens distortion parameters were also calculated and used for the rectification of the distorted images.

**Figure 11:**
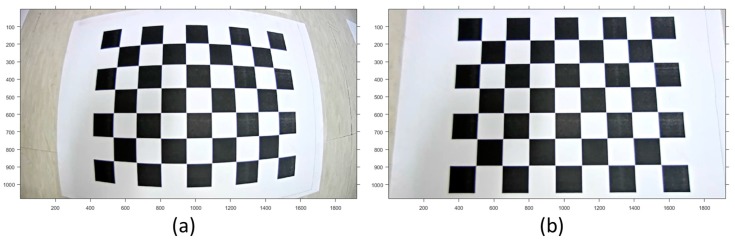
Result of image rectification with calibrated IOPs: (**a**) original image; (**b**) rectified image.

Figure 11 shows the image rectified with the calibrated parameters.

As well as the calibrated values, the precision of each parameter also could be quantified by the a priori standard deviation. Since the focal length and principal points can have a significant effect on the results of the boresight and lever-arm calibration, the precision of the calibration should be checked previously. As a result of the camera calibration, the precisions of the calibrated focal lengths were below 0.001 mm and the precisions of the principal points were below 0.5 pixels. On the other hand, the residuals of the calibration also quantify the precision of the calibrated camera model. After the camera calibration, the RMS of the projection residuals could be released by 0.42 pixels.

### 4.2. Boresight and Lever-Arm Calibration Result

With the calibrated IOPs of each camera sensor and the features extracted from each sensing data and the reference point cloud, the boresight and lever-arm calibration of the camera and laser scanner of the MMS was conducted. Table 6 and Table 7 summarize the results of the boresight and lever-arm calibration.

As shown in Table 6, the precision of the calibrated boresight was about 0.1 degrees and the precision of the calibrated lever-arm was about 10 mm. As shown in Table 7, while the differences between the coordinates of the ground features projected onto the images and the coordinates directly extracted from the images were below 1 pixel, the discrepancy between the plane model and the points transformed using the estimated parameters was 12 mm.

Furthermore, to analyze the influence of plane geometry on parameter estimation, the number of the control planes were controlled and every combination were applied on the MMS calibration. Table 8 summarized the results of applying the controlled datasets.

**Figure 12:**
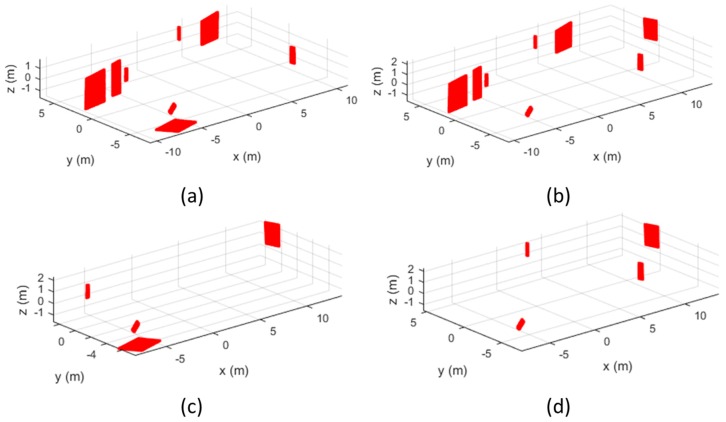
Example of good and bad geometries of control planes: (**a**) successful case with eight planes; (**b**) failed case with eight planes; (**c**) successful case with four planes; (**b**) failed case with four planes.

As shown in Table 8, when more than 9 control planes were used for the MMS calibration, the precisions of the lever-arm parameters could be achieved by 15 mm. However, when a smaller number of planes were used, the uncertainty of the calibration increased and precisions of the estimated parameters also decreased. In particular, even though eight control planes were used, the success rate was only 69%. Meanwhile, just with four control planes, the combination of 10% could achieve precision of 15 mm. This result implied that not only the number of control features but also their geometry must be carefully considered when designing the calibration site. Figure 12 illustrates the examples of good and bad geometries of control planes for precise calibration.

Using the control features represented in Figure 12a,c, the precision of estimated lever-arm parameters could be achieved by 8 mm and 12 mm, respectively. On the other hand, as shown in Figure 12b, the orientations of control planes also played an important role on the calibration. Since there were no ceiling or floor plane, the iterative least square adjustment could not estimate appropriate parameters.

**Figure 13:**
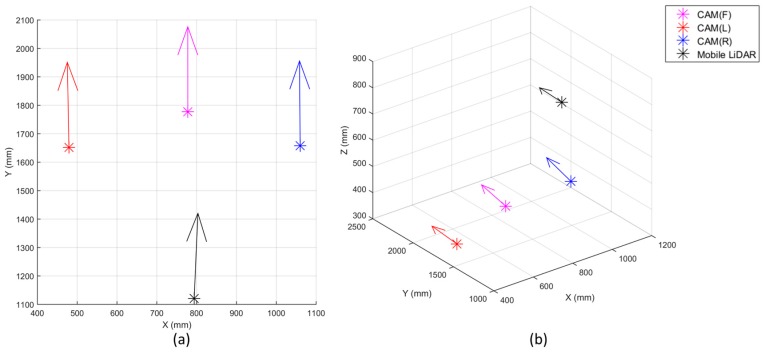
Boresight and lever-arm calibration results (In case of CAM(F), CAM(L), and CAM(R), the direction vectors in sensor frame are [0 0 1]. In case of mobile laser scanner, the direction vector in sensor frame is [1 0 0]): (**a**) 2-dimensional view; (**b**) 3D view.

With the boresight and lever-arm parameters, the relative coordinate systems of the sensing data were transformed into the single common coordinate system of the body frame. In Figure 13, the arrows represent where each sensor was and which direction each sensor oriented from the body frame.

**Figure 14:**
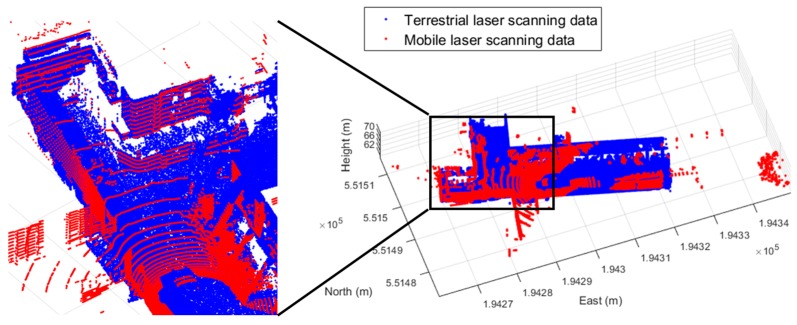
Georeferenced mobile laser scanning data.

**Figure 15:**
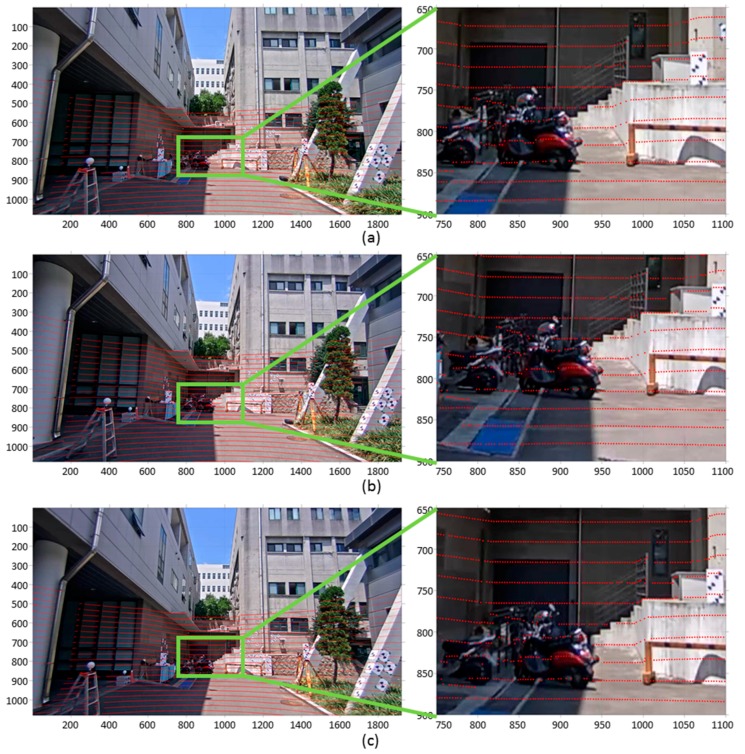
Projection of mobile laser scanning data into network video camera images: (**a**) CAM(F); (**b**) CAM(R); (**c**) CAM(L).

The data in the body frame can be transformed into the absolute coordinate system using the GNSS/INS data. Furthermore, with the parameters estimated from the calibration processes, the point cloud observed from the mobile laser scanner can be projected onto the captured images. Figure 14 represents the result of the transformation of the mobile scanning data into the Korean TM coordinate system. Figure 15 shows the result of the back projection of the mobile laser scanning data onto each image.

## 5. Discussion and Future Work

In this paper, the MMS combining the GNSS/INS, cameras and mobile laser scanner was developed and the boresight and lever-arm calibration of the MMS was conducted. The calibration approach based on the least squares adjustment using point features and plane features has been widely applied and continuously analyzed in existing researches for sensor calibration. However, for the adjustment, it is difficult to collect proper reference data for the calibration. In this regard, the utilization of the terrestrial laser scanner could be an alternative solution to efficiently achieve a reference dataset. Comparing with the total station and laser finder, which are generally used for collecting accurate positioning data, the terrestrial laser scanner could obtain a dense and precise point cloud and reference features for the MMS calibration.

**Figure 16:**
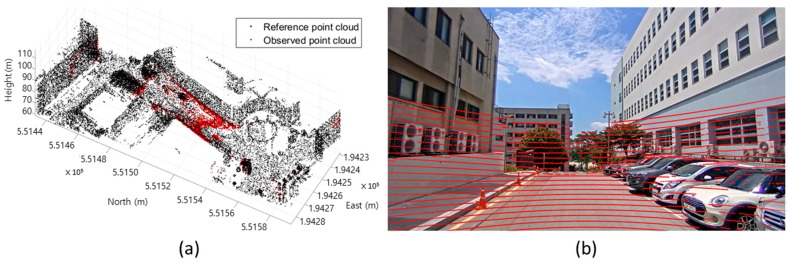
Operation of developed MMS under driving conditions: (**a**) georeferenced point cloud (red: observed by MMS, black: point cloud observed and georeferenced by terrestrial laser scanning data and GNSS); and (**b**) point cloud projected onto image.

Through the calibration parameters and GNSS/INS observation, the multi-sensor integration was conducted successfully and the point clouds observed by the mobile laser scanner were georeferenced into the absolute coordinate system or accurately projected onto the time-synchronized image (Figure 16).

**Figure 17:**
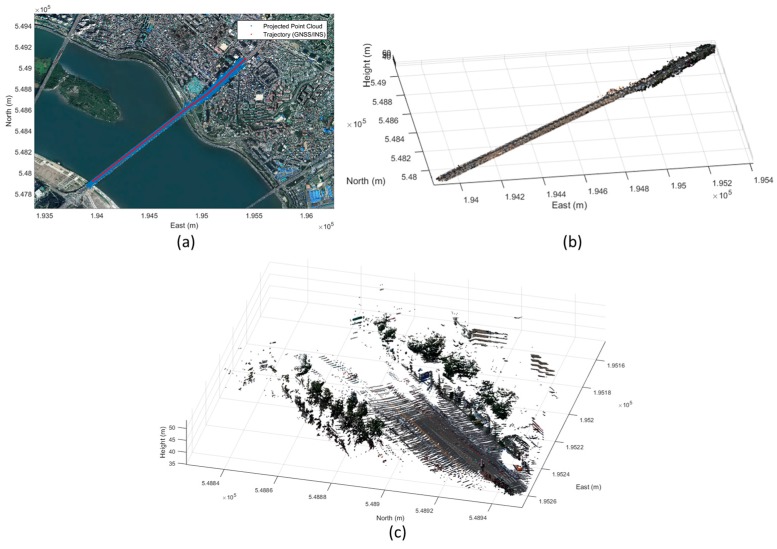
Point cloud generated by developed MMS: (**a**) point cloud projected on aerial orthophoto; (**b**,**c**) point cloud including color information.

**Figure 18:**
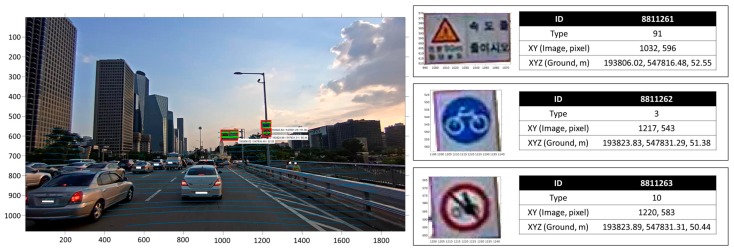
Example of 3D road sign mapping (white boxes: faces of people and registration numbers of cars are screened due to privacy, red box: road sign, green dot: points indicating road signs, blue dots, points projected onto the image).

The dataset continuously collected from the moving platform can be represented by two information formats: (1) the point cloud representing the 3D shape and color information of the observed objects (Figure 17); and (2) the 3D positional information of the objects extracted from the continuous images (Figure 18).

As shown in Figure 17, the point cloud observed from the MMS were directly georeferenced and could represent the 3D shapes of objects. However, the point cloud generated from the MMS was too sparse to extract the accurate road facility information. In particular, when objects were far from the MMS, the vertical point density of the generated point cloud became lower. For this reason, our research team alternatively designed the scheme of the road facility mapping based on image processing techniques. Moreover, as shown in Figure 18, the road facilities can be extracted from images and have absolute 3D coordinates. Our research team expects that the collected information of road facilities can be used as the basic data for the operation of autonomous cars. However, since the urban blockage of GNSS signals due to high and dense buildings significantly causes locational biases in the point cloud observed by the designed MMS, a proper Simultaneous Localization and Mapping (SLAM) technique to improve the stability and accuracy of observation should be developed and applied.

## 6. Conclusions

In this paper, the MMS combining network video cameras, mobile laser scanner, and GNSS/INS was developed and the effective procedure of the MMS calibration was proposed. By defining the reference features from a terrestrial laser scanning data, the precision of the boresight and lever-arm calibration could be achieved by approximately 10 mm and 0.1 degrees.

The main advantages of applying the terrestrial laser scanner to the MMS calibration problem are efficiency and maintenance. The mechanical analysis of each sensor is impossible for general users so a calibration process is required for the operation of an MMS. However, the observation of the accurate coordinates of the reference features is difficult and labor-intensive. In this regard, the application of the terrestrial laser scanner can significantly reduce the work time for the MMS calibration. Not only that, when the MMS users want to do the calibration again, the reference data constructed in the past can be applied for the new calibration process. For this reason, the calibration approach applying the terrestrial laser scanner can be a practical solution for general MMS users.

## Figures and Tables

**Table 1:** Specifications of the network video camera.

Model		AXIS F1005-E
Effective sensor size		1/2.8″
Focal length		2.8 mm
Field of view	Horizontal	113°
	Vertical	62°
Image size		1920 × 1200 pixels
Frame rate		60 fps

**Table 2:** Specifications of the mobile laser scanner.

Model		HDL-32E
Number of channels		32
Measurement range		Up to 100 m
Rotation rate		5~20 Hz
Accuracy		± 2 cm
Field of view	Horizontal	360°
	Vertical	−30.67~ 0.67°
Angular resolution	Horizontal	0.1~0.4°
	Vertical	1.33°
Laser		Class 1 903 nm Wavelength

**Table 3:** Specifications of the GNSS/INS unit.

Model	OxTS Survey+
Position accuracy	Up to 0.01 m
Velocity accuracy	0.1 km/h
Roll/pitch accuracy	0.03°
Heading accuracy	0.1°
Output rate	100 Hz
Size	234 × 120 × 88 mm

**Table 4:** Specifications of the terrestrial laser scanner.

Model		Focus 3D
Type		Amplitude-Modulated Continuous Wave (AMCW)
Measurement range		Up to 120 m
Scan rate		976,000 points/sec
Ranging error		±2 mm
Ranging noise		0.6 mm *
Field of view	Horizontal	360°
	Vertical	305°
Size		240 × 200 × 100 mm

* at 10m-raw data, at 90% reflectance.

**Table 5:** Camera calibration results (precision: 1 σ).

Parameter (Unit)		Initial Value	Calibrated Value ± Precision		
			CAM(F)	CAM(L)	CAM(R)
Focal length (mm)		2.5	2.47 ± 0.0054	2.44 ± 0.0069	2.52 ± 0.0085
Principal points (pixel)	*x_p_*	0	−27.41 ± 0.70	0.47 ± 0.87	−49.65 ± 0.95
	*y_p_*	0	33.05 ± 0.96	59.03 ± 0.87	18.14 ± 0.87
Radial distortion (unitless)	*A*_1_ (×10^−1^)	0	−3.33 ± 0.017	−3.39 ± 0.021	−3.57 ± 0.03
	*A*_2_ (×10^−1^)	0	1.29 ± 0.014	1.43 ± 0.021	1.65 ± 0.03
	*A*_3_ (×10^−2^)	0	−2.47 ± 0.045	−3.13 ± 0.078	−4.15 ± 0.15
Decentering distortion (unitless)	*B*_1_ (×10^−4^)	0	−5.16 ± 0.73	−8.59 ± 1.00	−3.51 ± 1.21
	*B*_2_ (×10^−4^)	0	1.98 ± 1.50	3.29 ± 1.51	−5.71 ± 2.35
Projection residuals (pixel)	x-direction	-	0.47	0.36	0.50
	y-direction	-	0.36	0.32	0.42

**Table 6:** Boresight and lever-arm calibration results (precision: 1 σ).

Parameter (unit)		Calibrated Value ± Precision	Parameter (unit)		Calibrated Value ± Precision
CAM (F)	$x_{T}$ (mm)	776.82 ± 6.25	CAM (L)	$x_{T}$ (mm)	478.36 ± 6.13
	$y_{T}$ (mm)	1776.02 ± 5.72		$y_{T}$ (mm)	1650.72 ± 5.61
	$z_{T}$ (mm)	383.23 ± 4.12		$z_{T}$ (mm)	340.34 ± 4.42
	ω (degree)	−81.8879 ± 0.0712		ω (degree)	−87.2623 ± 0.0725
	φ (degree)	−0.0425 ± 0.0621		φ (degree)	−0.7647 ± 0.0303
	κ (degree)	179.2535 ± 0.0815		κ (degree)	−179.8742 ± 0.0853
CAM (R)	$x_{T}$ (mm)	1060.64 ± 7.50	Mobile laser scanner	$x_{T}$ (mm)	793.87 ± 1.26
	$y_{T}$ (mm)	1656.25 ± 8.32		$y_{T}$ (mm)	1120.07 ± 1.34
	$z_{T}$ (mm)	424.36 ± 5.75		$z_{T}$ (mm)	892.54 ± 7.38
	ω (degree)	−83.0442 ± 0.0641		ω (degree)	−0.2845 ± 0.0642
	φ (degree)	−0.2962 ± 0.0446		φ (degree)	5.2074 ± 0.0534
	κ (degree)	175.9012 ± 0.0671		κ (degree)	88.2112 ± 0.0113

**Table 7:** Projection residual and external check result of boresight and lever-arm calibration.

Sensor (unit)	Projection Residual		External Check Result	
	Mean	RMSE *	Mean	RMSE
CAM(F) (pixel)	0.00	0.44	0.23	0.65
CAM(L) (pixel)	0.00	0.65	0.15	0.85
CAM(R) (pixel)	0.00	0.62	0.35	0.77
Mobile laser scanner (mm)	11.58	12.99	10.97	11.58

* Root Mean Square Error.

**Table 8:** Precision of estimated parameter and external check result according to plane number.

Number of Planes	Number of Precise Cases/Number of Combination * (percentage)	Precision of Estimated Parameters (mm) **			External Check Result (mm) **	
		$x_{T}$	$y_{T}$	$z_{T}$	Mean	RMSE
3	0/120 (0%)	-	-	-	-	-
4	22/210 (10%)	5.53	3.92	11.08	15.71	15.86
5	76/252 (30%)	1.15	1.79	9.13	11.41	12.16
6	97/210 (46%)	3.15	1.93	9.48	9.33	11.65
7	66/120 (55%)	0.99	1.18	7.03	8.22	9.01
8	31/45 (69%)	1.31	1.48	7.53	10.24	11.28
9	10/10 (100%)	1.49	1.64	7.84	9.21	9.78
10	1/1 (100%)	1.26	1.34	7.38	10.97	11.58

* The cases have estimated lever-arm parameter precisions better than 15 mm; ** The cases have the highest precision among the estimated parameters.
